# Impact of Dripper Type and Irrigation Water Salinity on Soil Bulk Density, Growth, and Yield of Maize Crop

**DOI:** 10.3390/plants14050693

**Published:** 2025-02-24

**Authors:** Hussein R. Nayyef, Mohammed A. Naser, Hasanen S. AL-Laghawi, Ali R. Alhasany, Ali H. Noaema, Barbara Sawicka

**Affiliations:** 1 Department of Tissue Culture and Medicinal Plants Techniques, Shatrah Technical College, Southern Technical University, Basrah 61001, Iraq; hussein.razzaq@stu.edu.iq; 2 Department of Soil Sciences and Water Resources, College of Agriculture, Al-Muthanna University, Samawah 66001, Iraq; 3 Wasit Governorate Agriculture Directorate, Ministry of Agriculture, Wasit 52001, Iraq; hasanen.allaghawi@gmail.com; 4 Department of Field Crops, College of Agriculture, Al-Muthanna University, Samawah 66001, Iraq; ali-raheem2002@mu.edu.iq (A.R.A.); ali.algayashe@mu.edu.iq (A.H.N.); 5 Department of Plant Production Technology and Commodities Science, University of Life Sciences in Lublin, 20-950 Lublin, Poland; barbara.sawicka@up.lublin.pl

**Keywords:** irrigation water salinity, bulk density, plant height, electrical conductivity

## Abstract

This study hypothesized that alternating fresh and saline irrigation with different dripper types would optimize water use while minimizing negative effects on soil bulk density (*ρ*b) and maize growth. The field experiment was carried out to investigate the impact of the types of the dripper and the salinity of irrigation water rotation on the *ρ*b, maize (*Zea mays* L.) growth, and yield using two kinds of drippers (turbo and spiral) and two levels of irrigation water with different salinity ratios (low, symbolized by L) and (high, symbolized by H). Irrigation water was added into three rotations (L, H), (H, L, H), and (L, H, L). Soil *ρ*b increased by 22.63% under saline irrigation, while yield was 3.07% higher with turbo drippers compared to spiral drippers. The results displayed an increase in plant height, leaf area, and yield by using the (L, H, L) as compared to (L, H) and (H, L, H), respectively. These results suggest that alternating fresh and saline water could reduce freshwater usage by 50% while maintaining acceptable crop yields, making it a cost-effective solution for water-scarce regions.

## 1. Introduction

The phenomenon of global warming has negatively affected the availability of fresh irrigation water in south Iraq, which requires the use of effective irrigation methods with irrigation water of different drippers and salinities. Drippers vary in the way that dissipate energy and water flowing through them, and one of the most popular methods used in designing emitters is to rely on friction and resistance to the flow of water through long paths, swirling motion, or even a series of nozzles. Using irrigation water with relatively high salinity and low salinity alternately will reduce the accumulation of salts in the soil body while allowing for the compensation of half of the plant’s water requirements [1]. High-pressure and frequent irrigation according to water requirement was superior in plant height, biological yield, and maize (*Zea mays* L.) grain yield to low-drainage irrigation by 1, 5 and 21%, respectively, [2]. Previous studies have examined salinity effects on maize growth, but few have investigated the combined impact of water salinity and dripper type on soil bulk density (*ρ*b) and crop yield. Al-Asadi et al. [3] and Xiukang et al. [4] presented in their studies that Iraqi lands are exposed to long-term and inevitable problems, including soil salinity, which is considered one of the main problems that devastate the country’s agricultural future. Despite the dryness and salinity of the lands, some difficulties in obtaining integrated data regarding the quantity and quality of groundwater in Iraq are presented, and they have caused significant losses in proportions in Arable lands. Arable lands, as some competent authorities, through field studies, gave the percentage of unsuitable lands at 45 million hectares (ha), or 76% of the total area of Iraq, while the cultivated area was estimated at 6 million ha [5]. Thus, this study tests the hypothesis that the (L, H, L) irrigation scheme will mitigate soil degradation while optimizing maize yield relative to continuous saline or fresh irrigation.

The selected irrigation schemes simulate real-world water management strategies where fresh and saline water sources are available intermittently. Increasing the use of irrigation water salinity levels above (6 g L^−1^) leads to the accumulation of salts in high proportions in the soil, which in turn works to raise the values of the *ρ*b of the soil and thus reduce porosity, as well as reduce the saturated hydraulic conductivity. These changes affected the physical properties of the soil, which reduced the leaf area index and plant height of corn, as well as the yield, which decreased by more than 25% when irrigated with water and salinity of 6 g L^−1^ [6,7]. It was found that the use of saline irrigation water for corn crops at a salinity level of 3 g L^−1^ for a short period was not clearly effective, as less than a 20% decrease in yield was observed, and this experiment used water over a period of three years [8]. A field study was conducted in Korbal plain, Iran to investigate the effect of irrigation methods’ exploitation of wastewater on soil moisture and salinity distribution. Soil samples were taken (before and after irrigation) at three depths of 0–20, 20–40, and 40–60 cm. The results of statistical analysis showed that the salinity concentration at a depth of 0–20 cm in subsurface drip irrigation was 1.66 ds m^−1^, while the minimum salinity value at a depth of 20–40 cm in drip irrigation was 0.92 ds m^−1^. The use of wastewater in irrigation caused an increase in salinity levels in irrigated soil compared to freshwater, regardless of the amount of water used in irrigation [9].

Hussein et al. [10] and Albayati and Topak [1] found that the use of salty irrigation water in the irrigation process leads to an increase in *ρ*b values, and they attributed the reason to the dispersal and breakdown of small soil aggregates and clay particles, which works to bring them together and deposit them in the spaces within the aggregates, thus clogging those pores, forming semi-solid and compact layers, and this effect was more prominent in the surface layer. While Dorraji et al. [11] and Huang et al. [12] found that increasing the salinity of the irrigation water led to a decrease in the rates of growing roots and a decrease in their branches in sandy loam soil for maize crop, where values were recorded as 0.84, 0.77, and 0.56 g plant^−1^ when using irrigation water with a salinity of 1.50, 4.00, and 8.00 dS m^−1^, respectively. The use of irrigation water for maize crops with varying electrical conductivity (EC) of 3, 6, and 9 dS m^−1^ led to an increase in soil salinity as the salinity of the irrigation water increased. Thus, the rate of salinity levels increased with 0.2, 1.8, and 2.8 dS m^−1^, respectively, and this in turn led to a decrease in the dry weight of the maize plant by 33.66%. The yield also decreased from 3.95 Mg ha^−1^ to 1.87 Mg ha^−1^ [13,14,15].

Likewise, Li et al. [16] and Rajpar et al. [17] noted the use of three irrigation water treatments with different EC, including an irrigation water treatment with a salinity of 3.8 dS m^−1^, a treatment of mixing 75% fresh water with 25% drainage water, and a treatment of mixing 50% fresh water and 50% drainage water, which gave grain yields of 78.81, 80.00, and 66.4%. Thus, the use of fresh irrigation water was reduced by 25 to 50%, and it was replaced by drainage water, accompanied by an insignificant decrease in yield. One of the problems facing Iraq is a sharp decline in the level of fresh surface water coming from the Turkish side, which has led to a deterioration of the chemical and physical properties in agricultural soil, thus limiting the areas suitable for agriculture. This decrease in water quantities in the Tigris and Euphrates rivers prompted researchers to encourage the use of modern irrigation methods as well as to resort to the use of drainage water and groundwater. Since groundwater has a high EC, the rotation method of using fresh and drainage water was used to conduct the irrigation process. Therefore, the overall goal of this study was to rationalize irrigation water and identify how the research factors affect the growth and yield of maize crops in the conditions of the southern region of Iraq. The specific objective was to study the effects of the type of dripper and the salinity of irrigation water rotation on the bulk density, growth, and yield of maize using two types of drippers (turbo and spiral) and two levels of irrigation water with different salinity ratios.

## 2. Results

### 2.1. Soil Bulk Density

**Figure 1:**
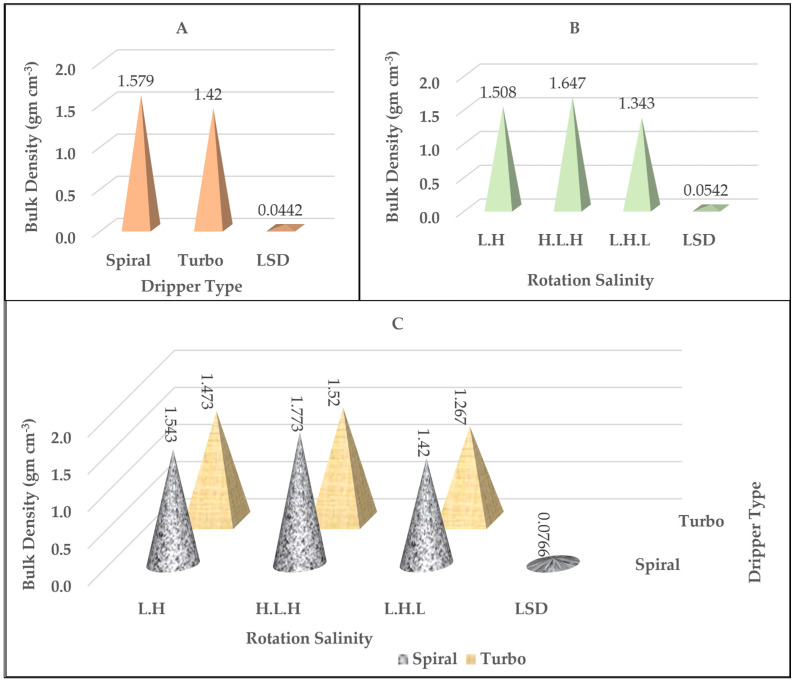
The effect of the type of dripper (**A**), the rotation salinity (**B**), and the interaction between them (**C**) on the soil bulk density values (g cm^−3^).

Table 1, Figure 1, and Table S1 (Supplementary Materials) show the effect of the study parameters (dripper types and salinity rotation) on the soil *ρ*b value, as it turns out that it increased significantly compared to its values before conducting the experiment (Table 2). The value of *ρ*b for the soil as in Figure 1A varied according to the variety of drippers used, as it reached its highest value when using the spiral dripper compared to the turbo dripper and for all cycles. The spiral dripper type gave the highest value of *ρ*b, amounting to 1.58 gm cm^−3^, compared to the turbo dripper, which recorded the best reading, which was 1.42 gm cm^−3^ [18,19]. The results from Table 1 and Figure 1B showed that there were significant differences between soil *ρ*b values according to the different salinity rotation used in the experiment, as the lowest value reached 1.34 g cm^−3^ in the L.H.L rotation, while the H.L.H rotation recorded the highest value and amounted to 1.67 g cm^−3^. Table 1 and Figure 1C showed that the lowest values recorded in their interaction with the salinity rotations (L.H., H.L.H, and L.H.L) were 1.47, 1.52, and 1.27 g cm^−3^ for the turbo dripper type compared to the spiral dripper overlapping with salinity rotation. It was noted from the results that the *ρ*b values decreased for the turbo dripper overlapping with rotations compared to the other dripper overlapping with the same rotations, and the percentages of decrease were 4.53, 14.26, and 8.66%, respectively.

### 2.2. Plant Height

**Figure 2:**
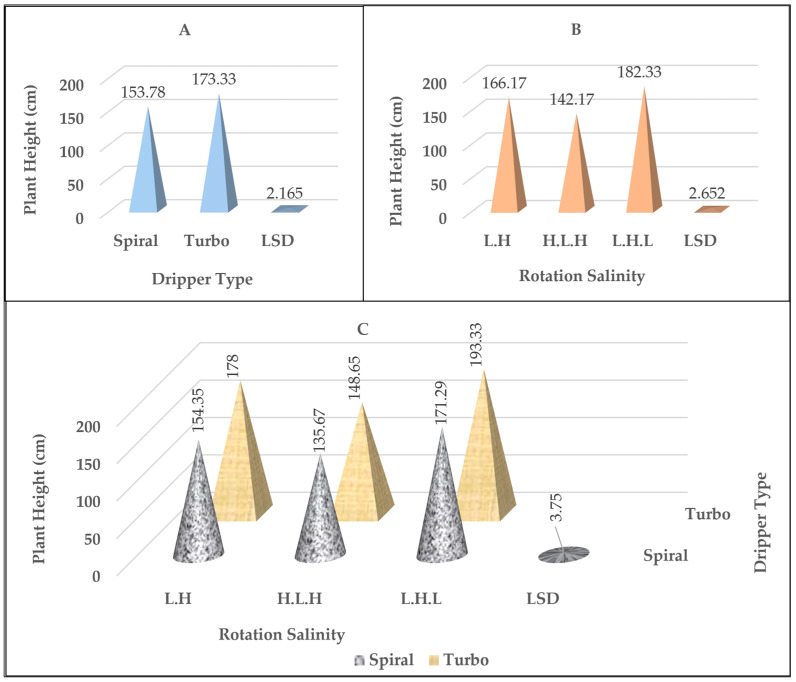
The effect of the type of dripper (**A**), the rotation salinity (**B**), and the interaction between them (**C**) on the values of plant height in cm.

It is clear that there was a significant effect of the dripper type factor on plant height (Table 1). When comparing the two dripper type treatments, it becomes clear that there was a significant difference in the plant height values as in Figure 2A. The treatment with the turbo dripper was superior and reached 173.33 cm compared to the spiral dripper and recorded 153.78 cm. The results presented in Figure 2B,C show the effect of the experimental parameters on the plant height values. There was a highly significant effect of the salinity rotation treatments in the use of fresh and drainage water overlapping with the type of dripper on the plant height values. As the salinity rotation treatment showed L.H.L overlapping with the dripper types, (spiral and turbo) were significantly superior compared to the treatments of L.H. and H.L.H, respectively. The plant height values ranged from 171.29 cm to 193.33 cm, compared to the rotation treatments overlapping with the dripper type, which ranged from 154.35 cm to 178.00 cm and 135.67 cm to 148.65 cm, respectively.

### 2.3. Plant Leaf Area

**Figure 3:**
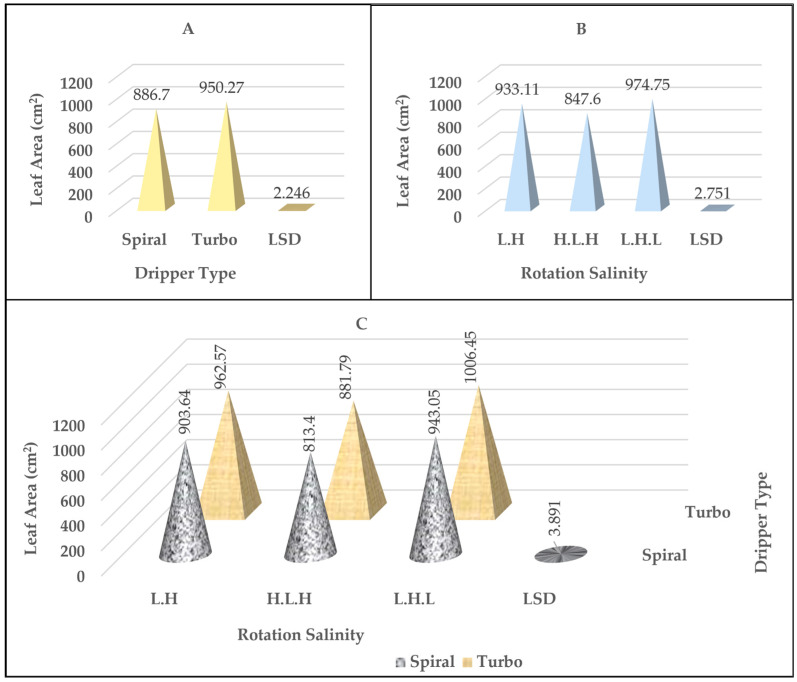
The effect of the type of dripper (**A**), the rotation salinity (**B**), and the interaction between them (**C**) on the leaf area values in cm^2^.

Table 1 and Table S3 (Supplementary Materials) show the results of the statistical analysis of the tabular F test. There was a significant effect for both the factors of dripper type and irrigation water salinity rotation and their interaction on the leaf area values. It is noted from Figure 3B that there was a significant superiority in the leaf area values for the salinity rotation treatment L.H.L, as it was recorded as 974.75 cm^2^, while the salinity rotation treatments L.H. and H.L.H gave the lowest values at an average of 933.11 cm^2^ and 847.60 cm^2^, respectively. Regarding changing the values of leaf area with the type of dripper, the results in Figure 3A show that there was a significant superiority for the turbo dripper. It gave the highest value compared to the spiral dripper, as the values, as an average for the two treatments, were 950.27 cm^2^ and 886.70 cm^2^ for the two drippers, respectively. The results of Figure 3C indicate that there was a significant effect resulting from the interaction between the type of dripper and the salinity rotation on the leaf area values. It turns out that the most significant differences were recorded between the turbo dripper type compared to the spiral dripper for all salinity rotations.

### 2.4. Grain Yield

**Figure 4:**
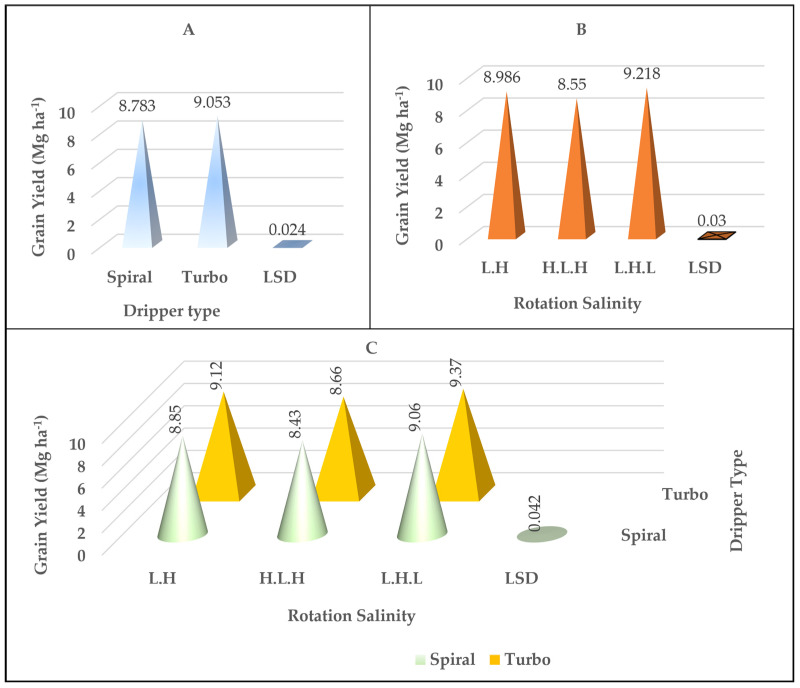
The effect of the type of dripper (**A**), the rotation salinity (**B**), and the interaction between them (**C**) on the values of grain yield in Mg ha^−1^.

The results showed that there was a highly significant effect on both the salinity rotation factors and the dripper type and their interaction on yield values (Table 1). The yield values and their distribution vary with the type of drippers. The results in Figure 4A show that there was a significant superiority for the turbo dripper, and the highest values were recorded at an average of 9.05 Mg ha^−1^ compared to the spiral dripper, where the values were an average of 8.78 Mg ha^−1^. There was significant superiority in the yield for the salinity rotation L.H.L treatment, which gave an average 9.21 Mg ha^−1^, while the salinity rotation L.H and H.L.H treatments recorded the lowest values, with an average of 8.98 Mg ha^−1^ and 8.55 Mg ha^−1^, respectively; Figure 4B. The (L, H, L) scheme resulted in a 10% higher yield than (H, L, H). Also, there was a significant superiority of the interaction between the salinity rotation and the dripper type in the yield values (Figure 4C). The L.H.L salinity rotation exhibited the highest significant differences in yield compared to the L.H. and H.L.H rotations across all drippers.

## 3. Discussion

The reason behind the high *ρ*b values for the H.L.H treatment is due to the relatively high levels of salinity in the soil, as well as the increase in the proportions of exchanged and absorbed sodium on the exchange complex. This leads to the deformation of soil aggregates and the dispersion of its clay particles, which in turn is considered a negative result represented by the blockage of pores and the decrease in their proportion and the increase in the *ρ*b of the soil, causing the destruction of the soil structure [20,21]. Also, the increase in *ρ*b observed under saline irrigation indicates compaction effects that may restrict root growth and water infiltration. The decrease in the *ρ*b values when using the turbine dripper may be due to the increase in the moisture content in the soil core, which in turn leads to an increase in the displacement of dispersed salts into soil aggregates, which encourages the root system to spread [22]. Our results from the effect of the dripper type and the alternation of salinity were consistent with the results by Yuan et al. [20].

The increased height of plants under the turbo dripper type may be attributed to enhanced uniform water distribution, reducing localized salt accumulation and improving root water uptake, thus enhancing crop height compared to the spiral dripper [23]. Increasing the height of plants in the L.H.L salinity rotation by interfering with the type of dripper (spiral and turbo) increases the efficiency of salts washing and improves the physical properties of the soil, which is reflected positively in the increase in vegetative growth, which is a response to the growth and spread of roots [24]. Meanwhile, in the treatments where the use of saline water increases, the plant height value decreased as a result of the water stress of the cultivated plant that exposed to saline water with high EC. As a result of the increase in the EC of irrigation water, this resultd in negative effects on the nutritional balance (mineral elements), and the vital processes that occur inside the plant such as photosynthesis (food production) and the inhibition of enzymes [25]. The increase in the salts in the plant is directly related to the increase in the concentration of dissolved salts in the soil solution, which in turn affects the physiology of the plant positively or negatively depending on the concentration and the circumstance. The positive effect is when the salts are at a moderate concentration, which in turn increases the efficiency of water use by reducing transpiration rates. While the negative effect of increasing the concentration of salts in the soil solution which increases salt stress as a result of increasing osmotic pressure, which reduces the plant’s ability to absorb irrigation water and thus leads to physiological drying even in the presence of water in the soil profile. The results reported by Hafez et al. [24] and Bouazzama et al. [25] supported our results that plant height increased with turbo dripper and salinity rotation L.H.L treatment.

The reason for the increase in leaf area values may be due to the use of rotation with low-salinity irrigation water. As a result, this led to improving the physical properties of the soil, such as increasing its aggregates, decreasing its consists, and increasing its porosity, as well as the density and spread of its roots, which was reflected in an increase in vegetation growth and leaf area values [26]. The superiority of the turbo dripper may be due to its increased discharge, leading to improved physical properties because of increased moisture content, and this is reflected in increased cell growth and division and then leaf area [27]. The use of a spiral dripper did not reduce soil salinity because of its decrease in discharge, which led to a decrease in leaf area due to high salt accumulation [28]. These results are consistent with what was found by Arbat et al. [28], who observed higher leaf area values for maize crops when irrigated with low-salinity water interspersed with drippers with good drainage efficiency.

The increased in grain yield was proportional to the increase in vegetative growth, which was reflected in an increase in the size of the root system and its spread, and thus had a positive effect on increasing the crop yield [29]. The increase in yield may be attributed to the result of using a rotation system in which the salinity ratio is reduced, which leads to improving the physical properties of the soil. This works to create a suitable growth environment for plants and ideal conditions for the growth of the root system and the expansion of the absorption area of water and nutrients as a result of low osmotic pressure as well as increasing the activity of beneficial soil organisms such as fungi and bacteria, which help in the decomposition of organic matter, providing the plant with the necessary nutrients, which is the site of grain accumulation [30]. The reason for the reduction in yield in the spiral dripper and the relatively high salinity irrigation water rotations is because of the decrease in soil moisture content, and the increase in the accumulation of salt levels in the zone was very close to the drippers. One of the problems with spiral drippers is that they are constantly clogged, especially when using salty water due to the deposition of salts and minerals in them. This leads to the inhomogeneous distribution of water to the plants, which leads to weak growth and low yield [14]. These results are consistent with what was found by Phullan et al. [30] and Guo et al. [14], which is that crop yields increase by performing appropriate irrigation according to water demands by using a quality dripper that resists clogging and can offer a reliable water discharge. The (L, H, L) scheme resulted in a 10% higher yield than (H, L, H), possibly due to better salt leaching during fresh irrigation cycles. Similar results were observed in wheat studies in North Africa, where alternating irrigation improved yield while reducing soil salinity buildup [19]. Prolonged saline irrigation may lead to permanent soil structure degradation, requiring additional leaching strategies for long-term sustainability.

## 4. Materials and Methods

### 4.1. Study Site

**Figure 5:**
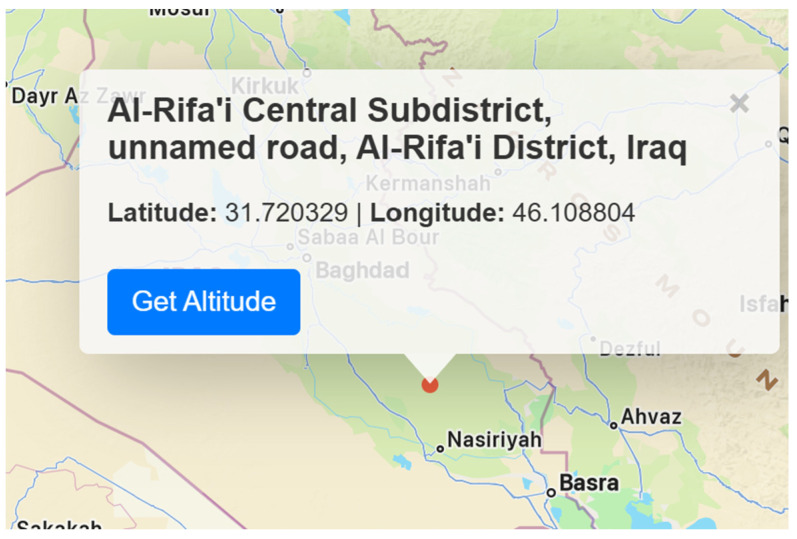
Study site map location.

This experiment was conducted in the field in Al-Rifai District-Dhi Qar Governorate, located on the geo-coordinates of latitude 31.7203291° N and longitude 46.1088035° E (Figure 5). There was no precipitation received during the maize growing season from 1 August to 30 November 2023 as presented in Table 2. Moreover, the average daily temperature was 22.7 °C and the average total monthly solar radiation was 438.9 W m^−2^ [31]. The average daily temperature during the crop growing season was higher than the average daily temperature for ten years, which was 18.6 °C, as shown in Table 2. The average total monthly solar radiation was higher than the average total monthly solar radiation for ten years, which was 354.5 W m^−2^.

### 4.2. Experimental Procedure

The soil used in this study was classified as silty clay loam (USDA classification). Before sowing, the soil was plowed and leveled to ensure uniform irrigation infiltration [32]. After completing the soil preparation operations, soil samples were taken to different depths (from 0 to 20 cm, 20–40, 40–60 cm) after digging a soil core at the experimental site. The soil samples were air-dried and passed through 2 mm sieves for the purpose of estimating some physical and chemical properties as shown in Table 3.

**Figure 6:**
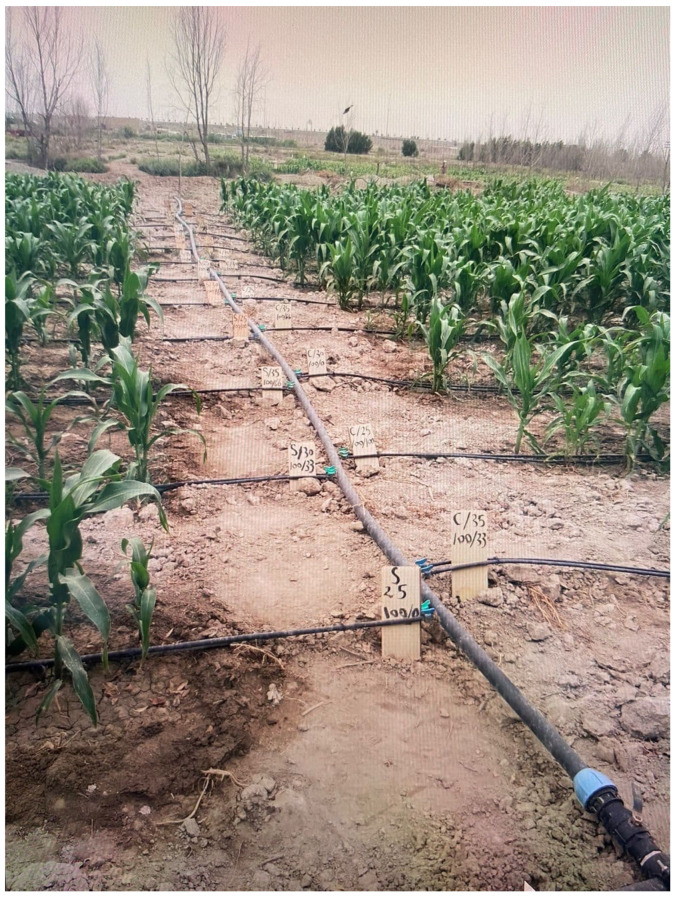
Field experimental plots of maize crop with drip irrigation system near the city of Rifai, Dhi Qar Governorate, Iraq.

The field experiment was designed using a Randomized Completely Block (RCB) design with three replications. A drip irrigation system was installed at the study site using a pump with a discharge capacity of 15 m^3^ h^−1^ for the purpose of raising irrigation water and pushing it into the pipes of the system with equal pressures after controlling it using the return water lock. Spiral and turbo drippers were chosen based on their reported efficiency in saline irrigation systems. The spiral dripper is characterized by its spiral design that can help to reduce the clogging of the openings and ensures a continuous flow of water. The main advantage of the spiral dripper is its design ability to regulate the flow of water as the water passes through a spiral channel that leads to its exit slowly, which reduces the opportunity of the dripper becoming clogged with sediment or dirt. The second type is the turbine dripper, which has an internal design similar to turbines, since it contains internal blades or channels that rotate as the water passes through them. This turbine design helps regulate the flow of water and decrease the water pressure to come out at a specific and constant rate. The system was operated, and the calibration process was conducted for the purpose of obtaining regular distribution and homogeneity (Figure 6). Irrigation was scheduled using a soil moisture sensor, where subsequent irrigation was performed when 50% of the moisture content was depleted at the field capacity. Two types of irrigation water were used: fresh water with the EC ranging from (1.35–1.6) dS m^−1^, and drainage water with an EC of (6.45–6.9) dS m^−1^. Six experimental treatments were distributed randomly in each sector so that the total number of experimental units was eighteen (Figure 6), which included two factors. Maize Barekat Jovain variety seeds were planted in the fall season on 28 July 2023 in holes at a depth of 4 cm on both sides of the field irrigation pipe at a distance of 5 cm from the dripper and at a rate of three seeds in each hole, while the distance between the holes was 20 cm on the same line. Maize is grown in Iraq in two seasons: a spring season at the end of February that continues until July, during which average temperatures in Iraq are relatively high, reaching 35–37 °C degrees, which negatively affects the pollination process and pollen grains, which in turn causes a decrease in production. The fall season, which begins at the beginning of August, during which the temperature is relatively high, but gradually decreases with the increases of stages growth until it reaches an ideal temperature that encourages the pollination process and the setting of grains in the cobs, thus increasing yield.

**Figure 7:**
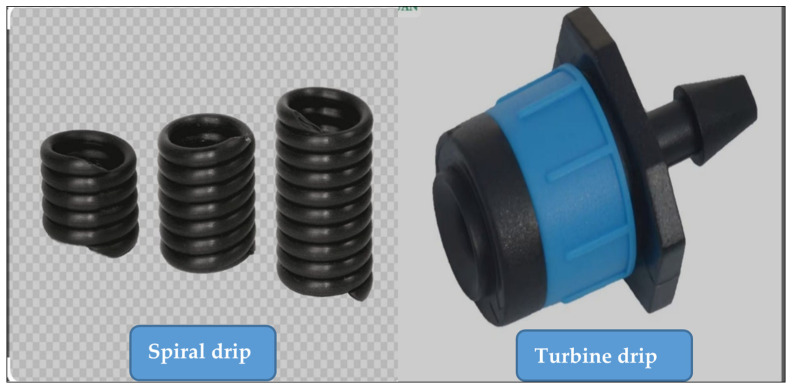
Types of drippers: spiral dripper (on the **left**) and turbo dripper (on the **right**).

The first factor was the types of drippers, which were spiral and turbo. The spiral dripper is an internal channel that takes the shape of a snail, and is characterized by a long water path with a small diameter ranging from 1 to 2 mm. The idea of its operation is to resist the flow of irrigation water, thus reducing the flow speed, achieving balanced pressure, and discharging the water in the form of water droplets. The turbo dripper is characterized by a vortex circular flow, which reduces the occurrence of sediment inside the water stream in the dripper, which increases performance in agricultural environments. It is also characterized by the self-regulation of pressure, which ensures equal distribution of water, with ease of disassembly and assembly, as well as ease of controlling the opening of the drippers (Figure 7). The second factor was the rotation of irrigation water salinity. The rotations of irrigation water salinity were as follows: A: Binary rotations (L and H), meaning low (L) salinity irrigation water (fresh water) with an EC of (1.35–1.6) ds m^−1^ was used in the first irrigation. While in the second irrigation, high (H) salinity water (drainage water) with (6.45–6.9) ds m^−1^ was used. Thus, the irrigation process was repeated using fresh water (L) in the third irrigation, and drainage water (H) in the fourth irrigation, and so on. B: Triple rotations (H, L, and H): In this rotation, drainage water (H) was used in the first irrigation, fresh water (L) was used in the second irrigation, and then drainage water (H) was used in the third irrigation. The irrigation started again with drainage water (H) used in the fourth irrigation, and fresh water (L) was used in the fifth irrigation, and the drainage water (H) was used in the sixth irrigation, and so on. C: Triple rotation (L, H, and L): The first irrigation was conducted using fresh water (L), the second irrigation was conducted with drainage water (H), and then the third irrigation was used with fresh water (L). The irrigation started again with fresh water (L) used in the fourth irrigation, and drainage water (H) used in the fifth irrigation, and then fresh water (L) used in the sixth irrigation, and so on.

This sequence was selected based on previous studies showing that intermittent saline irrigation can reduce salt buildup while maintaining soil moisture levels. Soil *ρ*b was measured using the core sampling method at depths of 20 cm and 40 cm. Plant height and leaf area measurements were taken at the silking stage (R1), when maize plants exhibit peak biomass accumulation. Maize yield was measured after harvesting on 14 December 2023, and the growing season length (GSL) from the date of planting to maturity was 140 days. Thus, FAO classification was between 400 and 600, and the maize hybrids maturity classified as late [33]. A cost–benefit analysis was conducted to compare the economic viability of each irrigation treatment based on water savings and yield output.

### 4.3. Statistical Analysis

Statistical analysis was conducted to examine the effect of the experimental factors on the studied traits, considering the effect of each individual factor on the trait, and then the effect of the two factors overlapping on the scientific trait studied in the experiment. The experimental data were statistically analyzed according to analysis of variance (ANOVA) for all studied traits using the statistical program Genstat, version 10.30E 2010 [34,35]. Statistically significant differences were calculated between the averages of the coefficients, with the least significant difference at the 0.05 level.

## 5. Conclusions

The water scarcity and desertification that our country Iraq is going through, in particular, and the world, in general, has negatively affected the environment and climate in Iraq. Therefore, researchers in this field must find solutions and proposals, including benefiting from available drainage water in southern Iraq without negatively affecting the properties of the soil and agricultural crops. Thus, as we mentioned previously in the research, the system of rotation of using fresh and drainage water for irrigation contributed to rationalizing the use of fresh water by 50% in the rotation (L.H.) and gave encouraging results in both the physical properties of the soil and in the vegetative system of plants as well as in the yield, compared to the alternation (L.H.L). This study has proven the possibility of using relatively high saline water (drainage water) as an available resource in southern Iraq, which can be mixed with fresh water and is considered a possible irrigation strategy for corn crops, especially in areas exposed to scarcity of irrigation water as is the case in the southern regions of Iraq. This approach could be tested by researchers in sandy soils, where water retention properties differ significantly from clay-dominated soils. Also, further studies should examine the impact of saline irrigation on microbial soil health and nutrient availability. For optimal results, farmers in arid regions should adopt (L, H, L) irrigation cycles to balance water efficiency and crop performance.

## Figures and Tables

**Table 1:** Analysis of variance for tabular F values for the values of the studied characteristics.

S	df	Bulk Density (g cm^−3^)	Plant Height (cm)	Leaf Area (cm^2^)	Yield (Mg ha^−1^)
Dripper type	1	62.22 **	387.20 **	3801.59 **	573.29 **
Rotation Salinity	2	74.57 **	551.41 **	5270.62 **	1207.60 **
D × R.S	2	6.81 **	11.11 *	7.03 *	5.33 *

R.S = Rotation salinity. D = Dripper type. ** Significant at 0.01 level. * Significant at 0.05 level.

**Table 2:** Summary of annual total precipitation (mm), average temperature (°C), and average total monthly solar radiation (W m^−2^) through the maize growing season for historic 10-year average weather data and the 2023 season for the study site.

Season	Aug.	Sep.	Oct.	Nov.	
	Precipitation (mm)				Total
2023	0.0	0.0	0.0	0.0	0.0
Ten Year	1.8	0.2	1.1	2.4	5.5
	Average Temperature (°C)				Avg.
2023	35.0	25.8	15.8	14.1	22.7
Ten Year	27.7	22.3	15.8	8.5	18.6
	Total monthly solar radiation (W m^−2^)				Avg.
2023	738.0	615.3	387.3	15.0	438.9
Ten Year	602.6	497.9	287.6	29.8	354.5

**Table 3:** Summary of physical and chemical soil attributes for soil samples were taken at depths of 0–20, 20–40, and 40–60 cm.

Properties		Soil Depth		
		(0–20 cm)	(20–40 cm)	(40–60 cm)
Bulk density (g cm^−3^)		1.36	1.37	1.39
Weighted diameter (mm)		0.13	0.11	0.10
Sand (g kg^−1^ soil)		158.00	150.00	149.00
Silt (g kg^−1^ soil)		476.00	478.00	475.00
Clay (g kg^−1^ soil)		366.00	372.00	376.00
Soil texture		Silty clay loam	Silty clay loam	Silty clay loam
pH		7.80	7.20	7.10
Total carbonate (g kg^−1^)		325.69	290.42	286.39
Organic matter (g kg^−1^)		3.35	2.63	1.98
EC ds m^−1^		2.67	3.85	3.97
Particle density (g cm^−3^)		2.55	2.55	2.55
Porosity %		51.00	50.00	48.00
Field capacity %		31.37	32.84	33.19
Ca^2+^	(dS L^−1^)	13.86	14.87	15.48
Mg^2+^	(dS L^−1^)	8.06	10.08	10.57
Na^+^	(dS L^−1^)	50.35	60.46	68.77
K^+^	(dS L^−1^)	1.79	2.69	2.92
HCO_3_^−^	(dS L^−1^)	3.34	3.08	3.01
SO_4_^−2^	(dS L^−1^)	17.46	17.84	17.96
CL^−^	(dS L^−1^)	57.93	61.74	63.87

pH = Soil reaction, EC = electrical conductivity, ds m^−1^ = desimines per meter, and g kg^−1^ soil = gram per kilogram.

## Data Availability

The original contributions presented in this study are included in the article and Supplementary Material. Further inquiries can be directed to the corresponding author.

## Supplementary Materials

The following supporting information can be downloaded at: https://www.mdpi.com/article/10.3390/plants14050693/s1, Table S1: Effect of the interaction between the type of dripper and rotation salinity on the bulk density values (g cm^−3^); Table S2: Shows the effect of the interaction between the type of dripper and rotation salinity on the values of plant height in cm; Table S3: Shows the effect of the interaction between the type of dripper and rotation salinity on the leaf area values in cm^2^; Table S4: Effect of the interaction between the type of dripper and rotation salinity on the values of grain yield in Mg ha^−1^.
