# Supplementary material for: Impact of Dripper Type and Irrigation Water Salinity on Soil Bulk Density, Growth, and Yield of Maize Crop
Source: Plants (Basel). 2025 Feb 24;14(5):693. doi: 10.3390/plants14050693 (PMC11902005; doi:10.3390/plants14050693)
Supplement: Supplementary file 1 [file plants-14-00693-s001.zip › plants-3465523-supplementary.pdf]

Supplementary Materials

Table S1. Effect of the interaction between the type of dripper and rotation salinity on the bulk density values (g cm^{-3}).

Emitter type	Bulk density (g cm^{-3})					
	Irrigation water salinity rotation				Average type of emitters	
	L.H	H.L.H	L.H.L			
Spiral	1.543	1.773	1.420		1.579	
Turbo	1.473	1.520	1.267		1.420	
LSD ET*IWS		0.0766		LSD E.T	0.0442	
Average Irrigation water salinity	1.508	1.647	1.343			
		0.0542				

Table S2. Shows the effect of the interaction between the type of dripper and rotation salinity on the values of plant height in cm.

Emitter type	Plant height (cm)				
	Irrigation water salinity rotation				Average type of emitters
	L.H	H.L.H	L.H.L		
Spiral	154.35	135.67	171.29		153.78
Turbo	178.00	148.65	193.33		173.33
LSD ET*IWS		3.750		LSD E.T	2.165
Average Irrigation water salinity	166.17	142.17	182.33		
		2.652			

Table S3. Shows the effect of the interaction between the type of dripper and rotation salinity on the leaf area values in cm^2.

Leaf area (cm^2) Emitter type	Irrigation water salinity rotation			Average type of emitters
	L.H	H.L.H	L.H.L	
Spiral	903.64	813.40	943.05	886.70
Turbo	962.57	881.79	1006.45	950.27
LSD $_{ET*IWS}$		3.891	LSD $_{E.T}$	2.246
Average Irrigation water salinity	933.11	847.60	974.75	
		2.751		

Table S4. Effect of the interaction between the type of dripper and rotation salinity on the values of grain yield in Mg ha^{-1}.

Yield (Mg/ ha^{-1}) Emitter type	Irrigation water salinity rotation			Average type of emitters
	L.H	H.L.H	L.H.L	
Spiral	8.85	8.43	9.06	8.78
Turbo	9.12	8.66	9.37	9.05
LSD $_{ET*IWS}$		0.042	LSD $_{E.T}$	0.024
Average Irrigation water salinity	8.98	8.55	9.21	
		0.030		
